# A shifting mutational landscape in 6 nutritional states: Stress-induced mutagenesis as a series of distinct stress input–mutation output relationships

**DOI:** 10.1371/journal.pbio.2001477

**Published:** 2017-06-08

**Authors:** Ram P. Maharjan, Thomas Ferenci

**Affiliations:** School of Life and Environmental Sciences, University of Sydney, Sydney, New South Wales, Australia; Massachusetts Institute of Technology, United States of America

## Abstract

Environmental stresses increase genetic variation in bacteria, plants, and human cancer cells. The linkage between various environments and mutational outcomes has not been systematically investigated, however. Here, we established the influence of nutritional stresses commonly found in the biosphere (carbon, phosphate, nitrogen, oxygen, or iron limitation) on both the rate and spectrum of mutations in *Escherichia coli*. We found that each limitation was associated with a remarkably distinct mutational profile. Overall mutation rates were not always elevated, and nitrogen, iron, and oxygen limitation resulted in major spectral changes but no net increase in rate. Our results thus suggest that stress-induced mutagenesis is a diverse series of stress input–mutation output linkages that is distinct in every condition. Environment-specific spectra resulted in the differential emergence of traits needing particular mutations in these settings. Mutations requiring transpositions were highest under iron and oxygen limitation, whereas base-pair substitutions and indels were highest under phosphate limitation. The unexpected diversity of input–output effects explains some important phenomena in the mutational biases of evolving genomes. The prevalence of bacterial insertion sequence transpositions in the mammalian gut or in anaerobically stored cultures is due to environmentally determined mutation availability. Likewise, the much-discussed genomic bias towards transition base substitutions in evolving genomes can now be explained as an environment-specific output. Altogether, our conclusion is that environments influence genetic variation as well as selection.

## Author summary

The importance of this study is in advancing our understanding of mutation supply—of the frequency that beneficial mutations arise in a population—in evolution and the contribution of stress-induced mutagenesis to this process. Evolutionary divergences, the emergence of antibiotic resistance in bacteria, and cancer in humans are all examples of processes powered by mutational DNA change. We show for the first time that stress conditions common in nature strongly influence the pattern of genetic variation and consequently the evolvability of traits dependent on particular mutations. More specifically, we show the nutrition-specific links between net mutation rate and type of stress and the significant plasticity of genetic variation in 6 environments. We reveal the nutrition-specific sensitivity of DNA repair and, most importantly, the demonstration that environmental spectrum differences impact evolution through constraints on mutations appearing at altered rates.

## Introduction

The notion of stress-induced mutagenesis (SIM) [1,2] has changed our perspective on the flexibility of mutation rates in organisms. The earliest evidence for SIM was that starvation can increase the supply of mutations, presumably increasing the capacity for adaptive changes and evolvability [1,3,4]. SIM is a collection of mechanisms observed in bacterial, yeast, and human cells, in which mutagenesis pathways are activated in response to adverse conditions, such as starvation or antibiotic stresses [5,6].

The detailed systems biology of mutation supply and its link to environmental states is still poorly defined, however. The most basic deficiency in understanding SIM is that neither the inputs nor the outputs of SIM are systematically defined. An analysis of what is meant by “stress-induced” (the input variation) and what is meant by “mutagenesis” (the output of subsets or all mutations) is essential for it to be clear whether SIM consistently involves the same environment-specific changes to DNA repair and mutation rates and the same mutational spectra with different stresses. An accurate modelling of evolution and detailed analysis of genomic signatures requires this level of information, and our study aims to provide clarity on this point.

**Fig 1:**
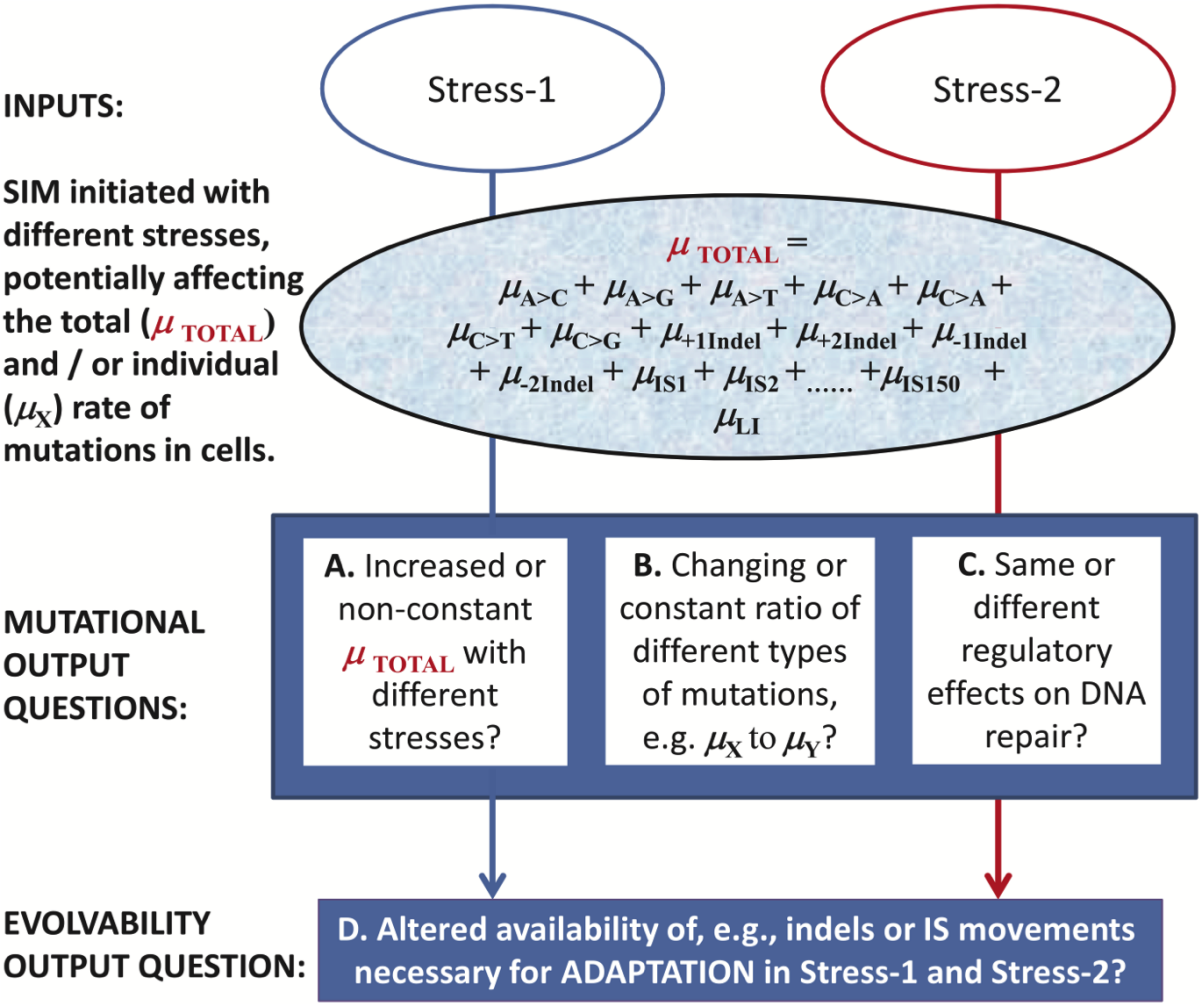
Unknown generic characteristics of stress-induced mutagenesis (SIM) in bacteria and other cell types. Different stresses are proposed to change the mix of mutations making up the total mutation rate (μ_**TOTAL**_ in the Figure). As such, different stress inputs can result in potentially distinct mutational outputs and effects on evolvability. The rates of the various types of mutation (μ_**X**_) defined in the text are in the Figure individually (e.g., base-pair substitution [BPS] mutation rates [μ_BPS_] are shown for each type of substitution such as the C to A mutation rate [μ_C>A_]). Examples of other rates are also included to underline the high number of individual rates when one considers, for example, that single base-pair indels (SI) involve +1, −1 base-pair insertion or deletions of different base-pair combinations. Boxes A–D show the questions addressed in this study. It should be noted that copy number changes with stresses are also possible, and diploid cells may also undertake different chromosomal rearrangements. These more complex changes are not addressed in the studies below.

Mutation availability is of obvious significance in the emergence of antibiotic resistance in bacteria or cancer in humans, as well as the stability of organisms in biotechnology and evolution in general. Both experimental and theoretical indications suggest that increasing the supply of mutations allows populations to overcome adaptive hurdles, such as those presented by antibiotic treatment [7–10]. Antibiotic-induced mutagenesis increases mutation rates and also changes the pattern of mutations [7,9]. To understand the full significance of SIM, the breadth of inputs into SIM need to be defined beyond stresses like starvation or antibiotics that are known to impact mutational processes [11]. In total, the majority of environmental and physicochemical stress effects on SIM are poorly understood. It is even uncertain whether the increased mutation rates in aging, starving bacterial colonies (the main initial evidence for stress-induced mutagenesis [1,3,4]) can be extrapolated to physicochemical stresses, generally. As indicated in Fig 1, there is no current information on the 4 output questions posed in boxes A. through D. on whether mutational processes, total mutation rates, individual mutation rates, and DNA repair respond similarly to distinct stressful environments common in the biosphere or even under standard laboratory culture conditions. What we know about DNA repair regulation in SIM is also limited to starvation and antibiotic effects. The identified effects are on mismatch repair down-regulation by starvation and antibiotics, as well as on the up-regulation of error-prone DNA polymerase by starvation [9,12–14].

The magnitude of the increased mutation rates and the magnitude of changes in mutational spectra is a particularly important question with SIM. As shown in Fig 1, the total mutation rate (μ_**TOTAL**_) [15] includes the 6 possible base-pair substitutions (BPSs), several single base-pair indels (SI, e.g., +1, −1 base-pair [bp] insertion or deletion of different base pairs), deletion and insertion indels >1bp (LI), and transpositions (with 10 different insertion sequence [IS] elements in *E*. *coli* K-12, for example). Approximations of the individual mutation rates, as well as amplifications, have been made [16], but these rely on data from various strains, selection environments, and laboratories. In this study, we devise a systematic analysis of whether the classes of mutation in Fig 1 fluctuate across environments and whether a composite mutation rate can really predict the outcome of evolution in different conditions.

Testing the environmental patterns of mutational rates and spectra is difficult because of the same experimental complexities that have bedevilled estimates of SIM [17]. The initial evidence for elevated mutation rates in bacteria under stress is based on estimating rifampicin resistance [1] or *lac* mutation reversion [3,4] in aging or starved colonies. These experiments have 2 problems. The first is that the environment in colonies on agar plates is complex, and the stress shifts over time [18]. The *lac* reversion assay is effectively carbon starvation [19], but the effects of other limitations have not been investigated on *lac* reversion. Secondly, the Lac and Rif assays, involving growth on lactose and rifampicin resistance phenotypes respectively, have been claimed to be compromised by the contribution of fitness and selection to the appearance of mutants on agar plates [16,17]. This problem is particularly important for Rif resistance [20], but the *lac* results have been reproduced in the absence of selection [21].

To avoid the problems of environmental fluctuation and selection, an analysis of the role of various stresses in bacterial mutagenesis required 2 essential ingredients. The first is the ability to fix environments to reproducibly compare both mutation rates and spectra. This challenge was solved by using bacteria that grow in controlled environments, in continuous (chemostat) cultures, at identical growth rates [22]. We controlled 5 resources essential for all forms of life, i.e., utilizable sources of carbon (C), phosphorus (P), nitrogen (N), iron (Fe), and oxygen (O). The availability of these is irregular in most ecosystems [23,24], including the human body, and bacteria like *E*. *coli* respond to these limitations through distinct patterns of gene expression [25]. Steady state limitation of C, O, N, Fe, or P was achieved by limiting the amount of glucose, oxygen, ammonium, iron, and phosphate salts in chemostats. Each of the nutrient-stressed environments fixed the specific growth rate to a constant 0.1 h^−1^, a 7-fold reduction compared to 0.7 h^−1^ with excess nutrients. At this reduced growth rate, bacteria are highly stressed and exhibit high concentrations of alarmones like guanosine tetraphosphate (ppGpp) and transcriptional stress controllers like RpoS [22].

A second requirement was an experimental system in which both the rate and spectrum of mutational types can be assayed without selection bias. Understanding genetic variation needs a comprehensive set of mutational types to be evaluated, beyond analyses that often focus just on BPSs [26,27]. Approaches like mutation accumulation (MA) experiments [28] are not environmentally controlled and somewhat biased because deleterious mutations in essential sequences are underestimated [29,30]. Some individual target gene analyses suffer from the above-mentioned fitness effects [17,27]. An example is the use of rifampicin-resistant *rpoB* mutations to analyse mutational spectra [31]; such studies are skewed by the large fitness differences and selectability of various alleles [32]. However, the use of a locus at a fixed chromosomal position eliminates the problem of variation in mutation rates between different chromosomal sites [33], especially when compared across environments. The method we adopt is to follow mutations in the *cycA* locus of *E*. *coli* resulting from cycloserine resistance (Cyc^R^) [30,34], which does not suffer the above problems. Cyc^R^ is conferred by a wide spectrum of loss-of-function mutations, including all 6 possible types of BPSs, different types of SIs and LIs, and IS transpositions across the entire length of *cycA*. Mutations in *cycA* conferring Cyc^R^ show negligible fitness effects ([30,34], so the environmental influences on mutations could be characterised without selection bias. The only disadvantage of this in-gene analysis is that stress-associated amplifications and larger chromosomal rearrangements associated with SIM [35] are not measured in this CycA study.

By using the above strategies, we demonstrate the remarkable plasticity of mutations in different conditions. We further show that the variation of mutation availability in different environments has detectable impacts on adaptation. The emergence of particular traits dependent on specific classes of mutations were especially affected by major changes in mutation spectrum. Environments thus provide alternative sets of keys to unlock various evolutionary pathways, thus impacting the role of genetic variation in evolution.

## Results

### Not all nutritional stresses elevate net mutation rates

**Fig 2:**
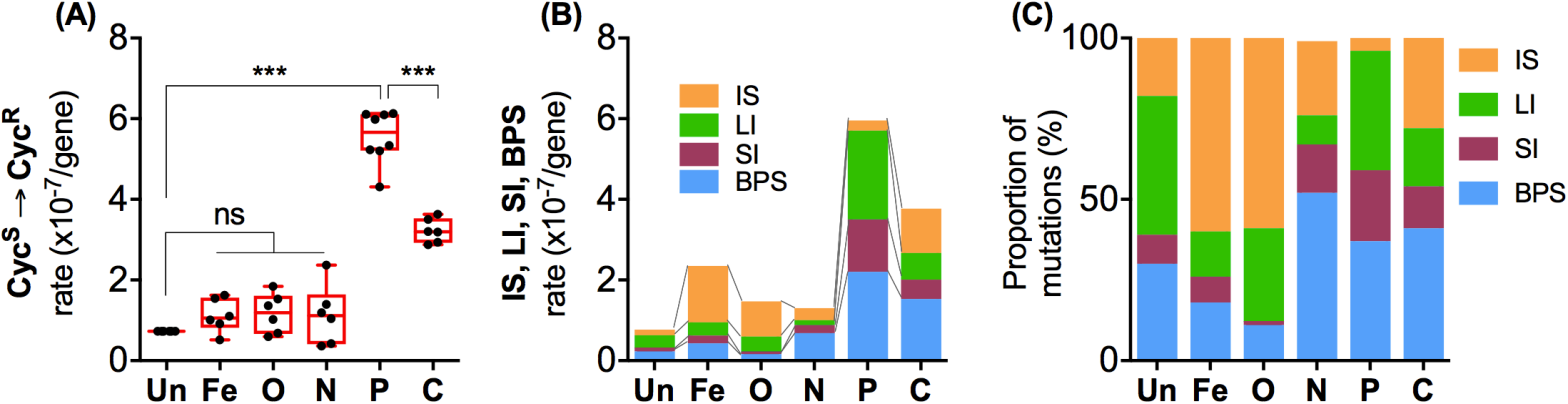
Mutation rate plasticity and fitness in 6 environments. (a) Mutation rates were calculated from the frequency of cycloserine resistance (Cyc^R^) mutants appearing in cycloserine-sensitive (Cyc^S^) cultures of *E*. *coli* for 6 replicate populations in each of 6 different nutritional states. Un (nutrient-unlimited) cultures were mid-exponential phase bacteria with a doubling time of 0.98 h, and iron (Fe) limited, oxygen (O) limited, nitrogen (N) limited, phosphorous (P) limited, and carbon (C) limited grew in chemostats with a fixed 6.9 h doubling time, as detailed in Methods. Box-and-whisker plots are shown, in which whiskers represent minimum and maximum values, the box represents top 75 and bottom 25 percentiles, and the horizontal line represents median value. Two-tailed *t* test *P* values were based on assuming 2-sample unequal variance. (b) Different colour bars represent the mean mutation rates of the 4 major classes of mutations (base-pair substitutions [BPS], single base pair indels [SI], deletion and insertion indels > 1bp [LI], and insertion sequence [IS] transpositions), based on the measurements in Fig 3D. Their contribution to overall mutation rates is shown in stacked bars for each environment. (c) The coloured bars represent relative contribution of BPS, IS, SI, and LI classes to the total mutation rate within each of 6 environments, which is shown as their proportion (%) on the basis of their frequencies in supplementary S1 Table. The numerical data for all parts of the figure are given in supplementary file S1 Data.

Five distinct nutrient limitations controlled in chemostats (of C, P, N, O, or Fe) and a nutrient-rich environment were used for investigating the link between nutritional stress and mutagenesis. In appraising mutations resulting in Cyc^R^ in the 6 environments, the first notable finding in Fig 2A is that *μ*_**TOTAL**_ (involving all types of mutations in *cycA* conferring Cyc^R^) is nonuniform across these conditions. The net mutation rate in nutrient-rich, rapid-growth conditions is low (*μ*_**TOTAL**_ = 0.73 ± 0.22 SD x 10^−7^ per locus per generation), similar to the value (0.65 x 10^−7^ per locus per generation) reported with batch cultures [34]. More surprisingly, the nutrient-rich mutation rate cannot be statistically distinguished from that under Fe, O, and N limitation (2-tailed *t* test, *P* > 0.05). It is remarkable that the 7-fold decreased growth rate under limiting Fe, O, and N does not result in a net SIM, i.e., elevated mutation rates due to “starvation” [1,3,4]. C and P limitation do induce SIM, and mutagenicity was highest with P limitation; C limitation was also higher than other conditions (*μ*_**TOTAL**_ = 5.6 ± 0.65 SD and 3.2 ± 0.29 SD x 10^−7^ per locus per generation, respectively; 2-tailed *t* test, *P* < 0.001 in both cases).

The observed 9- and 4-fold increases in P and C limitation respectively are notable because a 4-fold change in mutation rates can change evolutionary outcomes [36]. The *μ*_**TOTAL**_ spread we find in Cyc^R^ matches the range of mutation rates measured in several other ways with *E*. *coli* (see Table 1 for a detailed comparison). Differences in nutrition and environments may indeed explain the 10-fold span reported in these other environmentally less-controlled studies. In relation to the SIM discussions [1,3,4], our data suggests that plate-starved colonies increased mutation rates because of C or P limitation or both. Most importantly though, our observations suggest that not all nutritional stresses increase net rates; the nature of the starvation is more important than the generally decreased growth rate.

**Table 1:** Mutation rates in *Escherichia coli* obtained by different laboratories and methods.

Assay method and culture condition	Target gene or region	*μ*_BPS_ (per nucleotide site per generation)	*μ*_BPS_ (per genome per generation)	*μ*_TOTAL_ (per nucleotide site per generation)	*μ*_TOTAL_ (per genome per generation)	Reference/source
Mutation accumulation, colony transfer	Whole genome	1.9E − 10	0.0009	n.a.	n.a.	[37]
	Whole genome	n.a.	n.a.	2.2E − 10	0.0010	[28]
Luria Delbruck, batch	*lacI*	n.a.	n.a.	6.9E − 10	0.0030	[15]
Informatics	Various genomes	2.6E − 10	0.0012	n.a.	n.a.	[26]
	Various genomes	4.2E − 11	0.0002	n.a.	n.a.	[38]
Experimental evolution	Whole genome	8.9E − 11	0.0004	n.a.	n.a.	[39]
Luria Delbruck, batch	*cycA*	7.7E − 11	0.0004	1.1E − 10	0.0005	This study
Fe-limited chemostat	*cycA*	1.7E − 10	0.0008	2.9E − 10	0.0013	This study
N-limited chemostat	*cycA*	2.1E − 10	0.0010	2.7E − 10	0.0012	This study
O-limited chemostat	*cycA*	6.1E − 11	0.0003	1.5E − 10	0.0007	This study
C-limited chemostat	*cycA*	5.0E − 10	0.0023	6.6E − 10	0.0031	This study
P-limited chemostat	*cycA*	7.5E − 10	0.0035	1.0E − 09	0.0048	This study

To calculate per genome rates, the mutation rate in *cycA* was normalized to per nucleotide site or per genome by assuming Cyc^R^ phenotype is conferred by 273 sites in *cycA*. The number of sites (273*)* was based on a total of 543 BPSs (381 from this study and 181 from a previous study[30]) identified after sequencing more than 1879 sequences of Cyc^R^ clones in these studies.

**Abbreviations:** BPS, base-pair substitution; C, carbon; Cyc^R^, cycloserine resistance; Fe, iron; n.a., not applicable; N, nitrogen; O, oxygen; P, phosphorous; *μ*_BPS_, BPS mutation rate; *μ*_**TOTAL**_, total mutation rate.

**Fig 3:**
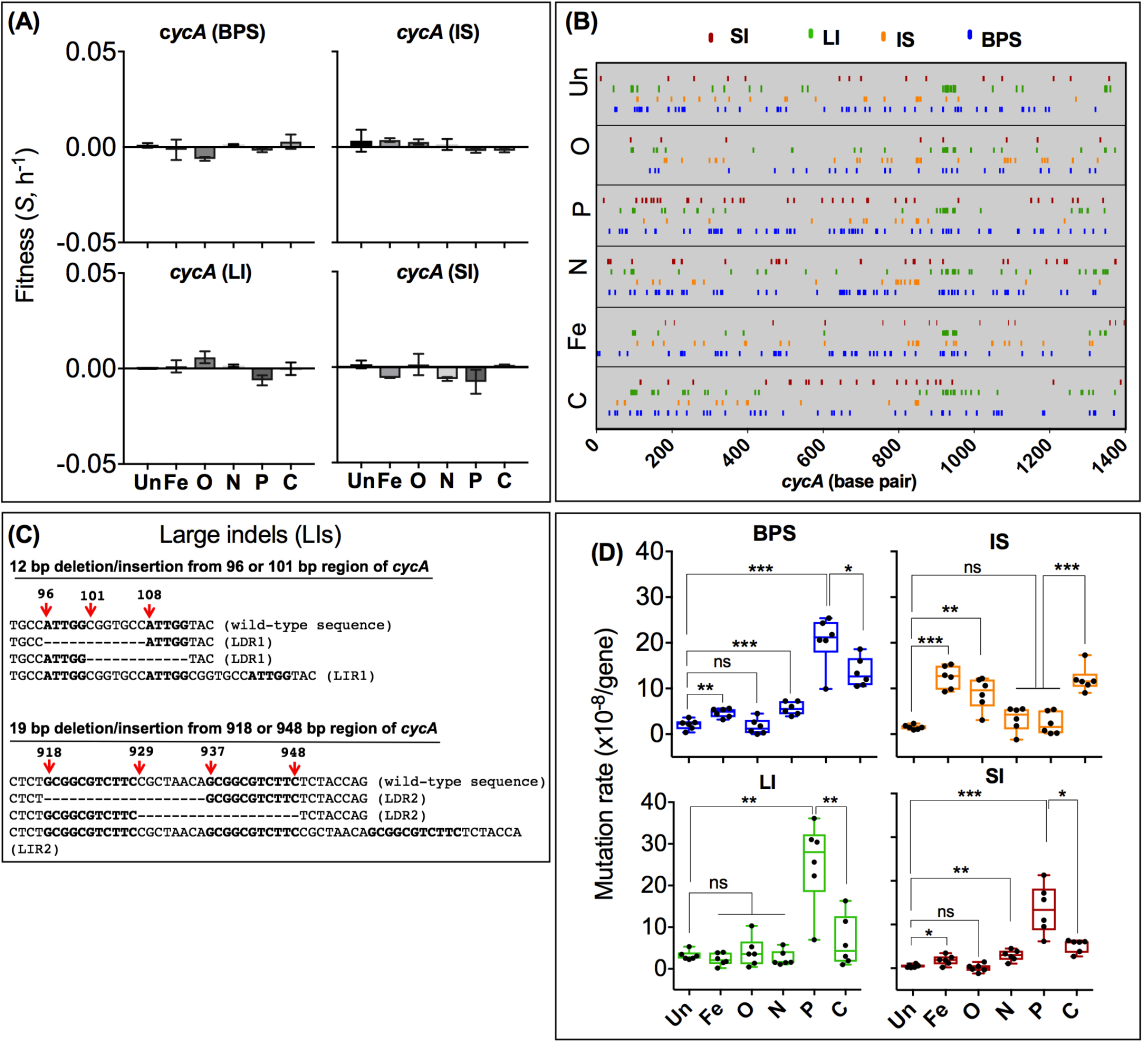
The distribution of fitness effects and mutation locations in *cycA*. (a) The fitness effect of cycloserine resistance (Cyc^R^) mutations are shown for examples of the 4 mutation classes: base-pair substitution (BPS, *cycA* G→T at position 298), insertion sequence (IS, *cycA* IS*150* at 848), deletion and insertion indels >1bp (LI, 19 base-pair deletion at 918), and single base pair indel (SI, -1G at 226), relative to the Cyc^S^ ancestor. Error bars are standard deviations from at least 2 replicate experiments. (b) The position of mutations in *cycA* in Cyc^R^ colonies. The plot includes sequence changes in 1,399 Cyc^R^ mutants, 228 from Un (nutrient-unlimited), 249 from iron (Fe)-limited, 234 from oxygen (O)-limited, 240 from nitrogen (N)-limited, 245 from phosphate (P)-limited, and 203 from carbon (C)- or glucose-limited cultures. (c) The location of large insertion and deletion mutations in *cycA*. Positions of deleted or inserted nucleotides are based on the sequence of *cycA* of wild-type *E*. *coli* MC4100 used in this study. Bold-typed nucleotides are short repeat sequences that we suspect promote insertion or deletion mutations. LDR1, a deletion of 12 bp at base positions 96–108 of *cycA*; LIR1, an insertion of 12 bp at base positions 96–108 of *cycA*. LDR2, a deletion of 18 bp in the 918–948 region of *cycA*; an insertion of 18 bp in the 918–948 region of *cycA*. Because there are only a few insertion mutations at region 1, LIR1 is combined with other deletion and insertion indels > 1bp (Other-LIs), which occurred across *cycA* as shown in Fig 4 in the main text. Locations of Other-LIs are not shown here but can be found in Supplementary material, S1 Table. (d) Rates of the 4 major classes of mutations (BPS, SI, LI, and IS transpositions) in 6 to 8 replicate cultures. Individual points and statistics of measured mutation rates in replicate cultures are shown for each class. The plots and statistics are presented as described in (Fig 2A). The numerical data for all parts of the figure are given in supplementary file S1 Data.

The observed higher mutation rate in C and P limitations was not due to increased fitness and enrichment of Cyc^R^ mutants. As shown in Fig 3A, all 4 major classes of mutation-conferring Cyc^R^ cause negligible fitness effects in all 6 environments, so these mutations were unlikely to be significantly enriched or eliminated in our experiments. The mutation rates were followed by sampling over only 72 h (see Methods), further limiting fitness effects on mutational frequencies and avoiding the potential distortion of population structures by later sweeps and hitchhiking in chemostats [40].

### Mutation spectra as environmental variables in SIM

To investigate whether nutritional SIM is associated with a uniform mutational spectrum in a series of distinct stresses, we sequenced the entire length of *cycA* in 1399 Cyc^R^ mutants arising after 72 h in the 6 conditions (see supplementary S1 Table for all mutants). Sibs were excluded by including only 1 example of each mutation from each culture. All the mutations were single mutations in *cycA*. The 4 major classes of mutation (BPS, SI, LI, and IS) are distributed throughout the *cycA* gene, and no particular BPS or SI hotpots were evident in the linear map depicting all detected mutations in *cycA* in the 6 nutritional states (Fig 3B). Many LIs were more localised due to repeat sequences in *cycA* shown in Fig 3C, but their frequency could still be environmentally compared.

We then estimated mutation rates independently for the BPS, SI, LI, and IS classes in all 6 conditions. The mutation rates for each type of mutation were estimated from their frequency in PCR-sequenced Cyc^R^ colonies, and the overall mutation rates for the *cycA* gene in each environment. The individual rates for each class in each environment are shown in Fig 3D. When rates for all classes were stacked, it became clear that *μ*_**TOTAL**_ based on sequencing (Fig 2B) is consistent with *μ*_**TOTAL**_ based on Cyc^R^ (Fig 2A). The contribution of each mutational class to the *μ*_**TOTAL**_ is, however, highly nutrition-specific (Fig 2C). Prominent differences include the IS mutation rates (*μ*_**IS**_), which are 8- and 6-fold higher in Fe and O limitations, respectively, than the *μ*_IS_ = 1.5 ± 0.51 SD x 10^−8^ per locus per generation in nutrient-rich conditions (Fig 3D, 2-tail, *P* < 0.01). Remarkably, the *μ*_IS_ differences occur between conditions that show indistinguishable *μ*_**TOTAL**_ rates (Fig 2A, 2-tailed *t* test *P* > 0.1). Another major difference was the *μ*_BPS_ = 20.4 ± 5.5 SD x 10^−8^ per locus per generation in P limitation, which was 10-fold higher than in the nutrient-rich condition (2-tail *t* test *P* = 0.0003). An even greater divergence was seen with single base-pair indel mutation rates (*μ*_SI_); *μ*_SI_ in P limitation (13.4 ± 5.58 SD x 10^−8^ per locus per generation) was more than 150-fold higher compared to *μ*_SI_ in O limitation (2-tail *t* test, *P* = 0.002). The LI rate (*μ*_LI_) also varied markedly in the 6 conditions (Fig 3D).

**Fig 4:**
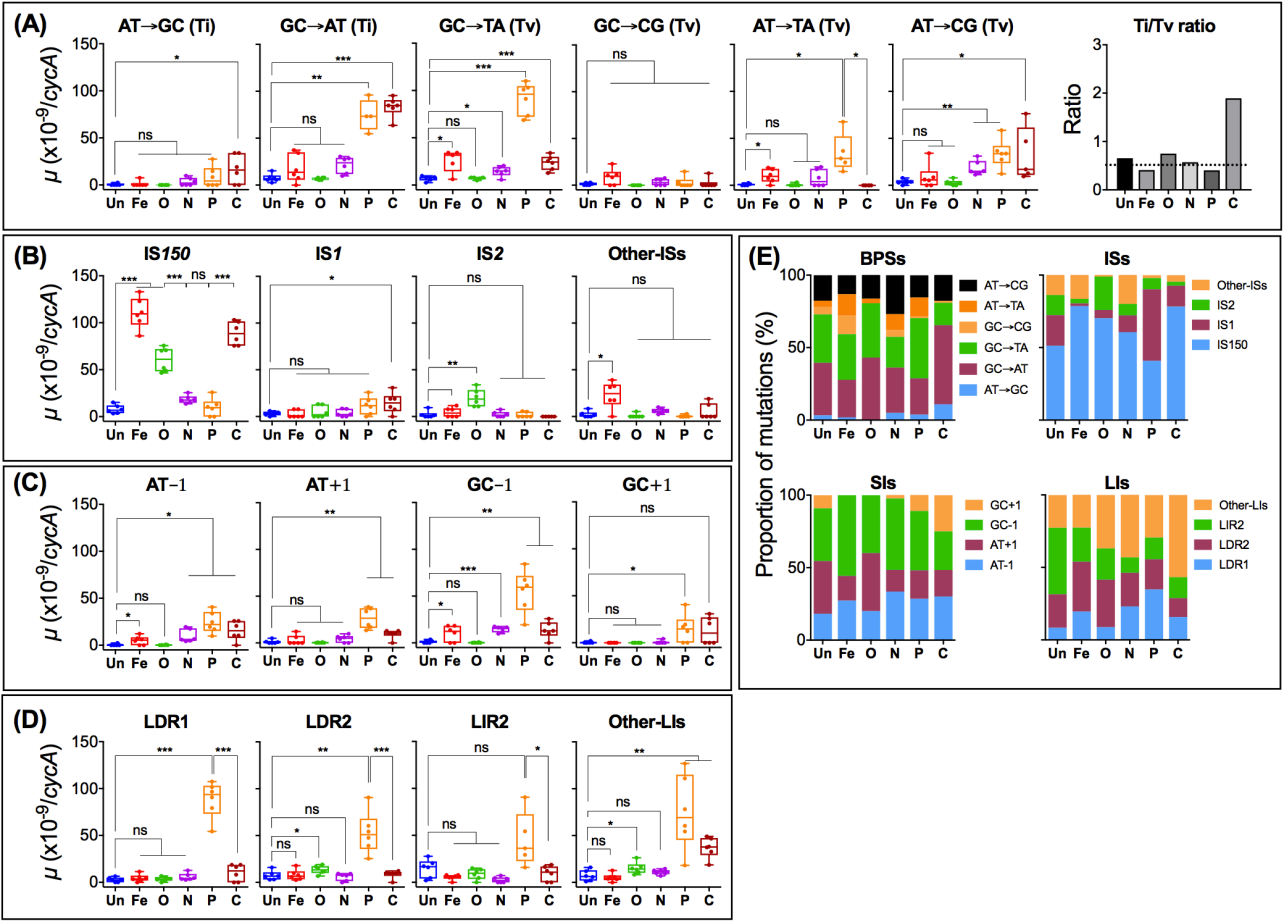
Mutation rates of 16 individual types of mutation in the 6 nutritional states. In all parts of the figure, the sample points, box-and-whisker plots and the symbols for the *P* values have the same properties as defined in Fig 2A. The environments are also as defined in Fig 2. (A) The mutation rates measured for each of the 6 different base-pair substitution (BPS) possibilities in 6 environments. The plot on the right summarizes the transition to transversion (Ti/Tv) substitution ratios amongst BPS changes in the 6 environments. (B) The transposition rates in *cycA* involving the insertion sequence (IS) elements shown; the plot “other ISs” includes the sum of all other rare IS movement rates. (C) The rate of 4 possible single base-pair indels (SI) in *cycA* with the loss or gain of 1 base pair (bp). (D) Deletion and insertion indels >1bp (LI) rates. See Supplementary S1 Table for the nature and position of LIs in *cycA*. (E) The coloured bars in each of the 4 panels represent the relative contribution of the individual mutations shown to the BPS, IS, SI, and LI classes within each of 6 environments. The bars represent the percentage contribution of each individual type to each class. The numerical data for all parts of the figure are given in supplementary file S1 Data.

As shown in Fig 4, nutritional effects on spectra are even more evident **within** each BPS, SI, LI, and IS class. Notably, 3 limitations (of O, N and Fe) that cause limited increases in BPS rates in Fig 4 contained a >10-fold variation in rates of all 6 individual BPSs (Fig 4A). P limitation showed the highest cumulative BPS mutation rate (*μ*_BPS_), and this was mainly due to GC→TA transversions and GC→AT transitions. Mutation rates for these substitutions were 10- to 13-fold higher than in nutrient-rich conditions (2-tailed *t* test, *P* < 0.001 in both cases). C limitation also resulted in a high BPS rate but mainly due to a GC→AT transition. We even found base changes that became extremely rare in particular environments; for example, GC→CG and AT→TA changes were below our detection limit in all 6 parallel cultures under O and C limitation, respectively.

An entirely unanticipated consequence of the shifts in BPS patterns is a uniquely different transition to transversion (Ti/Tv) ratio in 1 environment (Fig 4A). The Ti/Tv ratio is 1.9 in C limitation only but is closer to the random value of 0.5 with other limitations (Fig 4A). The strikingly high ratio in the C-limited environment resembles that commonly found between diverging genomes [41].

We found that 6 different IS elements conferred Cyc^R^ (IS*1*, IS*2*, IS*3*, IS*4*, IS*5*, and IS*150*) in *cycA*, but the elevated *μ*_IS_ under O, C, and Fe limitations was mainly due to IS*150* movement (Fig 4B and 4E). A remarkable consequence is that IS movements actually constitute the majority of mutations in O and Fe limitation (Fig 2B, Fig 2C). Another responsive IS movement was with IS*2*, which increased in O limitation compared to the unlimited condition (2-tail *t* test, *P* < 0.01). Our overall transposition rate (extrapolated by converting our *μ*_IS_ per locus rate to per genome rate; *μ*_IS_ = 4.9 ± 1.64 SD x 10^−5^ per genome per generation in the nutrient-rich environment) is 3-fold higher than the estimate of 1.5 x 10^−5^ per genome per generation in a mutation accumulation study [42]. The difference may be due to the deleterious effect of IS transpositions in MA experiments [29] or locus-specific bias in *cycA*. Aside from IS movements, other types of transposition events are subject to stress regulators [43], but we have no measure of these in the *cycA* experiments.

The single base-pair indel rates (*μ*_SI_) in P limitation were 40- to 150-fold elevated compared to nutrient-rich and O-limited environments due to 15- to 56-fold higher rates of all 4 types of SI (Fig 4C, 2-tail *t* test, *P* < 0.001). Likewise, significantly higher rates of 3 types of SI in C limitation (2-tail *t* test, *P* < 0.05) and 2 types of SI in N limitation (2-tail t-test, P < 0.05) differ from the less-extensive change in the 4 SI types among the nutrient-rich, O, and Fe limitation states (2-tail *t* test, *P* > 0.05).

The 4 LI events in Fig 4D were also nonuniform. A deletion of 12 bp at base positions 96–108 of the *cycA* gene (LDR1) was present in some mutants, while a deletion of 18 bp (LDR2) and an insertion of 18 bp (LIR2) in the 918–948 region of *cycA* (Fig 3C) was present in others. The total LIR2 rate was surprisingly prominent in the nutrient-rich condition (Fig 4E), which made LIs a prominent component of the mutation spectrum in nutrient-unlimited growth (Fig 2B and 2C). In contrast, deletion formation was especially elevated under P limitation.

These extensive spectrum findings in Fig 4 provide a new insight that nutritional states have a more general impact on stress-specific changes to DNA repair or mutagenesis than on overall *μ*_**TOTAL**_ mutation rates.

### How do environments cause a mutational bias?

**Fig 5:**
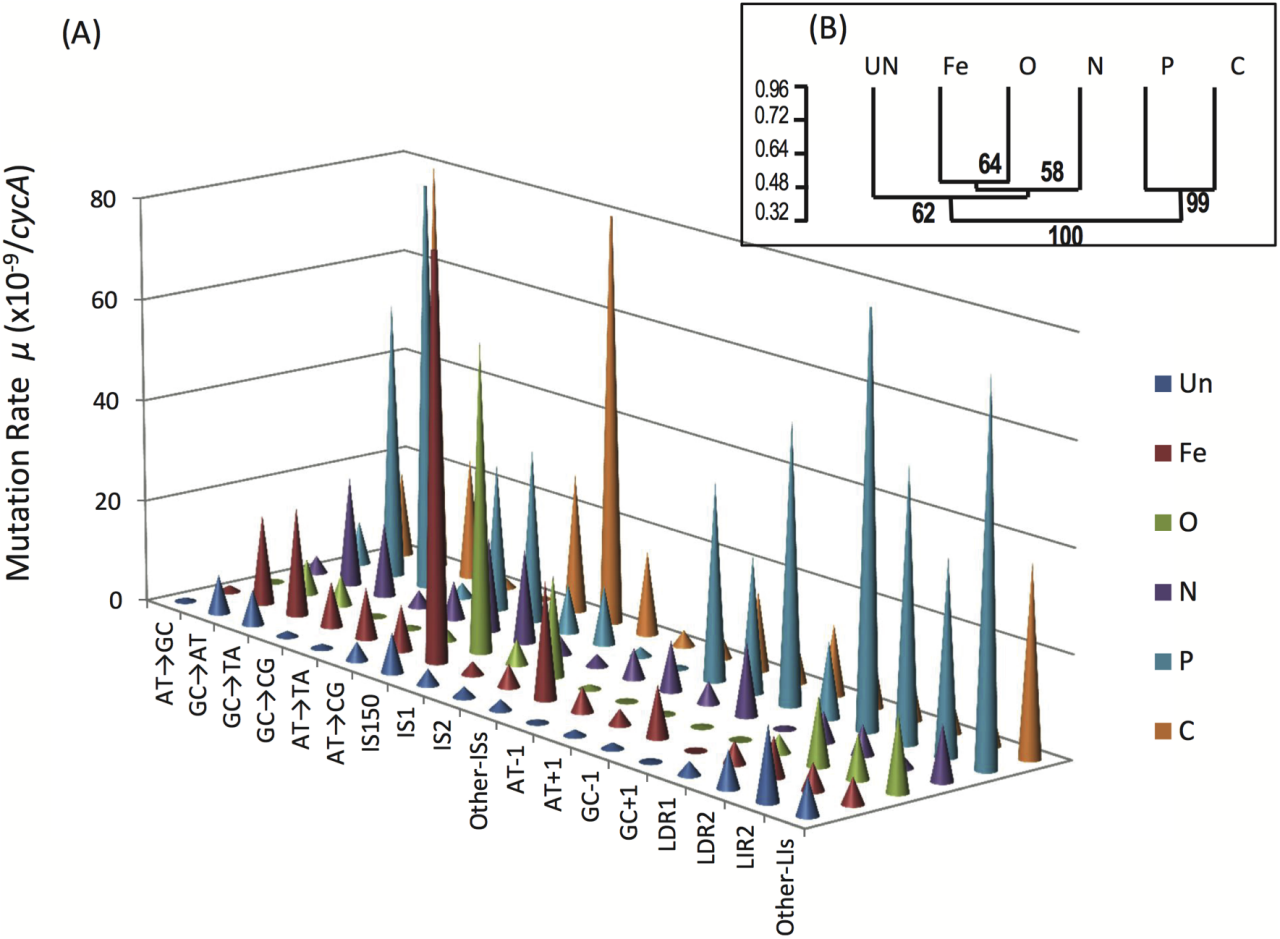
The mutational landscape in 6 nutritional states. (A) The landscape is based on the mean mutation rates of the 16 different types of mutation estimated in Fig 4A–4D plus 2 composite rates (other insertion sequences [Other-ISs] and other deletion and insertion indels > 1bp [Other-LIs]) in the 6 nutritional states. In (B), the relationship of mutational patterns is related by the Unweighted Pair-Group Method with Arithmetic Mean (UPGMA [44]). The bootstrap values were obtained from 1,000 replicate analyses.

The relationships between individual mutation rates and environments are summarized in a mutational landscape (Fig 5A). This highlights the considerable mutational pattern differences between environments. There is nevertheless a dichotomy in rate patterns between the nutrient-unlimited (Un), Fe-, and O-limited cultures and the more mutagenic P- and C-limited environments. This dichotomy is reinforced by the unweighted pair-group method with arithmetic mean (UPGMA) clustering in Fig 5B between sets of limitations, suggesting some environments result in more related mutagenic effects. Explaining these relationships is difficult, however. Physiologically, O and Fe limitation may show similarities if a reduction of Fe-initiated O radical damage is a cause of the pattern changes. On the other hand, the physiological relationship of O and Fe to N limitation is less obvious. The close clustering of the C and P limitations is also difficult to explain through their divergent patterns of gene expression [25].

**Fig 6:**
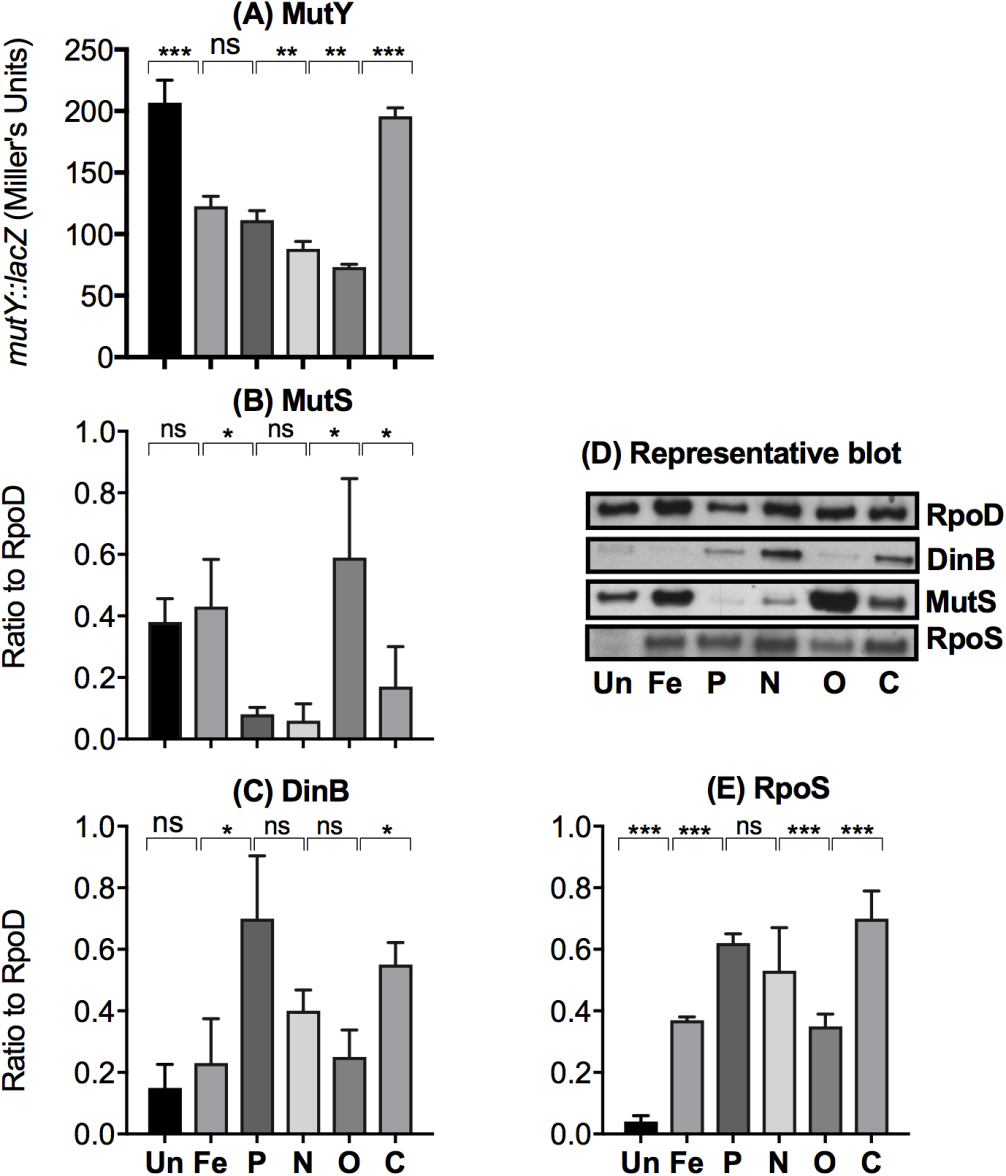
The effect of 6 nutritional states on three DNA repair systems and a regulator of multiple repair systems in *E*. *coli*. (A) MutY is an adenine DNA glycosylase active on GC→AT mispairs and corrects error-prone DNA synthesis past lesions, which are due to the oxidatively damaged DNA. Its expression was followed using a *mutY-lacZ* transcriptional fusion [46] in the 6 different nutritional conditions by following β-galactosidase activity. (B) The level of the DNA mismatch repair protein MutS, which corrects noncomplementary base pairs, was measured by immunoblotting relative to RpoD [50], a constant RNA polymerase component in *E*. *coli*. (C) The level of DinB (an error-prone DNA polymerase, pol IV, regulated as part of the SOS response) relative to RpoD was measured by immunoblotting [50]. (D) A representative western blot of the data in panels (B-E). (E) RpoS levels were analysed by immunoblotting relative to RpoD [50]. The error bars in plots represent the standard deviations from 3 independent experiments. *t* test *P* values were based on 2-tailed *t* test, assuming 2-sample unequal variance. *P < 0.05, **P < 0.01, and ***P < 0.001. ns, not significant. The numerical data for all parts of the figure are given in supplementary file S1 Data.

One clue to the complexity of the spectra is the unexpectedly intricate pattern of DNA repair system expression in each nutritional state (Fig 6). We quantitated a component of the ubiquitous mismatch repair system (MutS [9]), the SOS system (DinB involved in error-prone repair [19,45]), and MutY expression (involved in O-damage repair in *E*. *coli* [46]). The mismatch repair (MMR) and SOS systems and expression of *mutY* are not coregulated by nutritional factors, but all vary (Fig 6). There is no clear relation between repair levels and that of a global controller, RpoS (Fig 6E), known to affect both MutS and DinB [9,19]. Elevated RpoS in all limitations relative to unlimited conditions shown in Fig 6 should increase double-strand breaks according to [47], but we do not find correspondingly increased net mutation rates in some of the environments. Other inputs besides RpoS must control mutation processes. Another repair component, UvrD, was recently linked to stress [48] and is also likely to be subject to nutritional variation. The regulatory plasticity of repair systems may thus contribute to the basis of the complex shifts in mutation patterns seen in Fig 4. An indication of this is with the most mutagenic environment (P), which has the highest error-prone polymerase, equal lowest MutS, and reduced MutY repair. Of course, other influences on mutational spectra may contribute; the chromosomal structure and its superhelicity, subdomains, and DNA-binding proteins also changes in various environments [49]. Further analysis is needed to explain how environments impact on mutational spectra.

### The generality of spectral changes; is the cycA spectrum characteristic of other genes?

**Fig 7:**
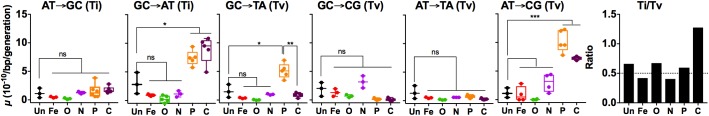
The effect of 6 nutritional states on base substitution patterns in *lacZ*. Mutation rates for each of 6 different possible base pairs, AT→GC, GC→AT, GC→TA, GC→CG, AT→TA, and AT→CG were assayed by using the tester *E*. *coli* strains CC106, CC102, CC104, CC103, CC105, and CC101, respectively. The transition to transversion (Ti/Tv) substitution ratios amongst all base-pair substitution (BPS) changes in the 6 environments are shown in the right panel. The environments and axis labels are as defined in Fig 2. Box-and-whisker plots are shown, in which whiskers represent minimum and maximum values, the box represents top 75 and bottom 25 percentiles, and the horizontal line represents median value. Two-tailed *t* test *P* values were based on assuming 2-sample unequal variance. In plots, * represents *P* < 0.05; ** represents *P* < 0.01, and *** represents *P* < 0.001. ns, not significant. The numerical data for all parts of the figure are given in supplementary file S1 Data.

**Fig 8:**
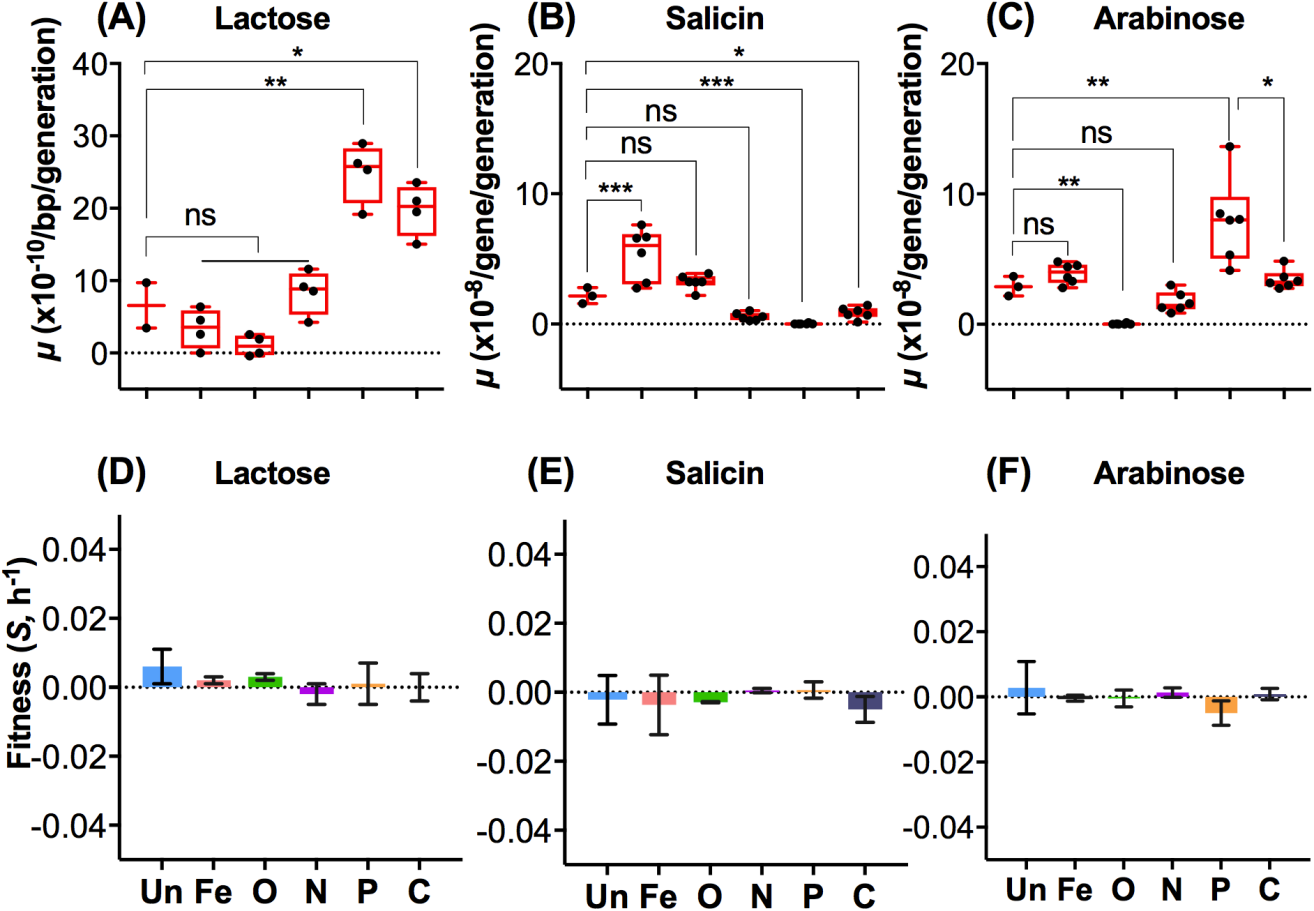
Environmentally controlled mutation availability influences the evolvability of new traits. Mutation rates of changes permitting growth on 3 different carbon sources, requiring different types of mutation, were measured. The adaptations required either a base pair substitution in *lacZ* (A, lactose), an insertion sequence (IS) transposition in the *bgl* operon (B, salicin), or an SI mutation with a +1 base-pair insertion in *araD* (C, arabinose). (D) The fitness of a Lac^+^ colony obtained from Lac^-^ was measured in the 6 different nutritional conditions against the ancestral Lac^-^. In (E), a Sal^+^ colony obtained from Sal^-^ was measured in the 6 different nutritional conditions against the ancestral Sal^-^. In (F), the fitness of an Ara^+^ colony was measured against the ancestral Ara^-^ strain in 6 conditions. Error bars are standard deviations from at least 2 independent experiments. *t* test *P* values were based on 2-tailed *t* test, assuming 2-sample unequal variance. In plots, * represents *P* < 0.05; ** represents *P* < 0.01, and *** represents *P* < 0.001. ns, not significant. The numerical data for all parts of the figure are given in supplementary file S1 Data.

There are suggestions that chromosomal position can influence rates of mutation [33] and other data suggests local context rather than location is important [51]. Our data in Figs 2–5 are based on the spectrum of CycR mutations in a single gene at position 95.44 min on the *E*. *coli* chromosome. Hence, it was desirable to test whether the spectral changes under the various stresses are similar at other chromosomal sites. We thus additionally followed, in the same 6 environments, the occurrence of 3 types of mutation at 3 other sites. These were in *lacZ* at 7.83 min (for all 6 types of BPS), *bgl* at 65.57 min (for IS movements), and *araD* at 1.42 min (for a single base indel). The data obtained are shown in Figs 7 and 8.

The analysis of *lac* BPS changes used the 6 strains developed by Cupples and Miller, each of which require a particular base change to revert to a Lac^+^ phenotype [52]. The 6 strains were each grown in the same 6 environments, and Lac^+^ isolates were enumerated after 72 h of chemostat culture, as for *cycA*. The data are resented in Fig 7. The reversion rates for each BPS were characteristic for each limitation, and AT→CG, GC→AT changes were strongly environment-dependent. For example, mutation rates for AT→CG and GC→AT are 9- and 3-fold higher in P limitation than in the nutrient-rich condition. This pattern was as observed for the *cycA* changes in Figs 3 and 4. As shown in Fig 8A, the sum of all 6 BPS rate changes in each environment was also environment-dependent and remarkably similar to the *cycA* pattern in Fig 3D. The individual BPS changes in *lacZ* also resulted in a similar Ti/Tv pattern as shown for *cycA* in Fig 4A, with glucose limitation having the highest Ti/Tv ratio. Comparing the *cycA* and *lacZ* results, it appears the stress-dependent BPS spectrum changes were both gene- and assay-independent.

A second test of the generality of environmental effects was on the differences in IS mutation rates in the 6 environments (Fig 4C). We used the mutational acquisition of the ability to use β-glucosides like salicin in *E*. *coli*, because this specifically requires IS transposition (several IS elements can be involved) near the *bgl* genes [53]. The expectation was that we see elevated IS movement in Fe and O limitation but not in P limitation, as with *cycA* (Fig 3D). Indeed, Sal^+^ mutations were commonest in O and Fe limitation and lowest in P limitation (Fig 8B, 2-tailed *P* < 0.001). There was a 10-fold range of *bgl* mutation frequencies in the 6 states. The Sal^+^ environment profiles match the IS spectra obtained from sequencing of *cycA*, except for C limitation (Fig 3D).

The third prediction was that large environmental effects on a +1 bp SI mutation in *cycA* (Fig 4C) should result in a commensurate rate of mutation at other loci. We tested the reversion to growth on arabinose in a mutant [54] with a 1 bp deletion in *araD* as an assay for the prevalence of a +1BP insertion. The reversion rates to Ara^+^ were measured in the 6 environments. As shown in Fig 8C, Ara^+^ reversion was also environment-sensitive. There was a >100-fold range of *araD* reversion rates. The strikingly clear result was that the frameshift change in *araD* was most prominent in P limitation but highly reduced during O limitation (Fig 8C, 2-tailed *P* < 0.001), altogether matching the SI pattern in Fig 3D. Sequencing of *araD* and PCR analysis for the *bgl* IS movement demonstrated that the mutational types were indeed a +1 frameshift and IS insertion, respectively.

In order to check whether the Lac^+^, Ara^+^, and Sal^+^ differences in Fig 8 were due to fitness advantages of mutants in the chemostats, the competitive fitness of Lac^+/-^, Ara^+/-^, and Sal^+/-^ mutations in each environment was tested to see if the mutations were differentially enriched in particular settings. The Lac^+^, Ara^+^ and Sal^+^ mutations were each near-neutral and the fitness of mutants is not a major difference in the 6 environments (Fig 8D and 8E). In the absence of fitness effects, mutation availability is thus the likely explanation for the uneven pattern of Lac^+^, Ara^+^, and Sal^+^ mutations across environments in Fig 8A–8C. The important consequence of these findings with the 3 sugars in the 6 environments is that the traits dependent on particular types of mutations are likely to emerge at different rates in different situations; this is also likely to apply to any adaptation that relies on specific types of mutation.

## Discussion

This analysis of nutritional states on mutation rates and spectra under stringently controlled conditions has produced results that challenge established assumptions on SIM and the randomness of mutations in genetic variation. The major surprise is the very distinct mutational mixes under 5 different forms of nutrient limitation at the same growth rate. This data suggests that mutational spectra are highly susceptible to the environment. The detected plasticity of all mutational types greatly extends mutation spectrum observations with individual stresses in various organisms [7,9,30,55]. Here we dealt with nutrient stress, but a systematic analysis of spectra with other physicochemical stresses such as pH and salinity at fixed growth rates are also called for to expand our understanding of how environments fix the scope of mutational genetic variation. A new study reinforces that the mix of mutations also changes with temperature [56], but the spectral changes were not presented in detail. Environmental effects on genetic variation are thus likely to be a general phenomenon.

Changes in total mutation rates with stress are long-argued but lacked a systematic analysis [1,3,4]. Our results indicate that nutritional stresses (and perhaps other environmental stresses) do not uniformly increase total mutation rates; only 2 of the 5 conditions tested significantly increased rates. One of these 2 conditions (carbon limitation) is likely to be a factor in the plate starvation assays used by others [1,3,4], so provide conditions where higher mutation rates are measurable. Altogether, our results point to the importance of fixing environments in studying mutational processes because variations to Fe, P, or aeration levels can occur at high cell densities in rich media, and this could bias mutation profiles. In addition, the results with C and P limitation, as well as studies showing how regulatory mutations change mutational processes [19,50,57,58] argue that stress effects on mutations are real, although very sensitive to environments. Another surprise was that P limitation was the most mutagenic, giving a 9-fold higher net mutation rate and the lowest level of DNA repair. It should also be pointed out that our results exclude selection effects on the *cycA*, *lacZ*, *bgl*, and *araD* mutations analysed in these experiments, eliminating one of the major problems present in earlier studies [16,17].

The generality of the spectral changes in our *cycA* study is vindicated by the changes also seen in the *lacZ*, *araD*, and *bgl* genes (Figs 7 and 8). Biased base-pair changes, single-base SIs, and IS movements are a general feature of different forms of nutrient limitation in the 4 genes. Furthermore, a new study published since submission has determined mutational spectra under anaerobiosis [59]. This too found the IS*150* difference to be the biggest single pattern change, as in Fig 4B. The small increase in total mutation rates (1.6-fold) in anaerobic mutation accumulation experiments [59] is also consistent with our data in Fig 2A. So we are confident of the generality of our findings within *E*. *coli* that environments have a strong impact on mutational spectra.

Our data provide an entirely new perspective of SIM. We find that **all** tested nutrient stresses impact on mutational spectra, and the availability of particular types of mutations. Likewise, **all** individual mutation types are stress-affected. The established description of SIM, as a process up-regulated by a stress response [5], covers only a part of an unexpectedly complex environment–mutation landscape. Stress, resulting in major decreases in growth rates, does not always equally increase mutation rates. We find novel situations in which stress regulators down-regulate particular mutational types and up-regulate others, but net mutation rates remain unchanged. Actually, the only universal mutational effect of suboptimal environments is that mutational spectra are shifted in all of the situations we studied.

It is interesting to speculate how the environmental influences may impact the evolution of bacteria, especially inside the mammalian habitat of *E*. *coli*. From our results, the implications for bacterial evolution are that acquisition of new traits needing IS transposition is much likelier in anaerobic (colon) or Fe-limited (body secretions) than P-limited situations. There is published evidence for this bias, because the majority of first-step bacterial adaptations in the anaerobic gut of mice are due to IS transpositions [60]. Another example of an anaerobic environment with unusually high rates of IS movement is in long-term stab cultures [61], and a new study has shown that transposition rates are also altered by medium agar concentration [62].

Another example of mutational bias likely due to a P-limited environment is in lungs [63]. SIs are most likely to occur in P-limited environments in our study. Indeed, there is evidence that SIs provide the majority of parallel adaptations in *Pseudomonas aeruginosa* in 474 longitudinal lung isolates [64]. Genome-wide analysis of these isolates also showed a much lower BPS/indel ratio (3.4) than observed in bacteria generally (19.3; [65]). The BPS/SI ratio with P limitation in Fig 4 was correspondingly low (1.6), whereas the highest ratio (32) was with O limitation.

The C-limited state is prevalent in the competitive intestinal environment of *E*. *coli*, and it is frequent in many parts of the biosphere. It is tempting to predict that the BPS bias to transitions we find to be prevalent under C limitation provides a plausible cause of the transition bias arising in evolving *E*. *coli* and other genomes [66]. Still, natural environments are often not single-nutrient limited so we cannot predict how more complex environments (e.g., involving both C and O limitation) affect mutation rates and spectra. Nevertheless, environmental causes may well contribute to the general transition bias in organisms [41], as shown in Fig 4A and Fig 7.

We do not have a detailed mechanistic explanation for shifts in spectra but the extensive environmental-mutational plasticity we find is associated with the equally remarkable patterns of DNA repair. The sheer complexity of the changes means that we cannot identify particular gene-regulatory networks as being impacted by particular nutritional states. RpoS and other networks associated with DNA repair may contribute [19,50,57,58], but there is no direct link between RpoS levels in Fig 6 and particular mutation patterns. It will need a comprehensive analysis of all repair functions to disentangle the spectrum differences.

In relation to evolutionary theory, these findings profoundly change our understanding of genetic variation in evolution. This is because spectral shifts in mutation availability occur even when net mutation rates do not. Since not all mutational types are able to initiate all fitness paths [67], the spectrum differences may well result in evolvability effects. The novel implication is that evolution provides alternative adaptive pathways, as proposed for evolutionary divergence [67]. Our inference is that models of evolution need to modify the historical concept of “mutation rate” as a parameter in evolution. Mutational heterogeneity as much as the net mutation rate may well influence evolvability; this notion is inconsistent with Neo-Darwinian assumptions that mutational effects have a minor role in evolution [68]. Especially relevant are situations in which the acquisition of new characters requires specific mutations rather than any random variation. For example, the spectra of IS and SI mutations in *bgl* and *ara* genes (Fig 8) indicates that IS and SI mutations are very limited in some environments but not in others. Altogether, our results support the view that the availability of mutations can be a factor in evolution and may drive the acquisition of new characteristics through supplying limiting mutations, called constraint-breaking mutations in recent models [68,69].

Another fundamental question arising from the mutation rate and evolvability differences is whether the mutation biases are random with respect to adaptation. Based on our results, we do not see a clear link between increased individual mutation types and their benefits in those same environments, as has also been shown for starvation-induced mutagenesis [70]. For example, SIs are not obviously beneficial under P limitation in *E*.*coli*, the environment in which they are so prevalent. Although SIs may be mostly deleterious, the 10-fold increase in BPSs under P limitation may potentiate alternative beneficial changes. The transition bias in C limitation does not offer clear benefits, nor does the strongly elevated IS transposition under anaerobic conditions. Nevertheless, some of many notions of mutational “chance” or “randomness” in evolution [71] are impacted by our findings. For now, our conclusion is that environmental biases do not provide directed mutations.

Finally, the implication of our results extends to all those important domains of biology and medicine where mutation availability and mutation bias have recognised roles. These include situations where particular mutations empower evolutionary divergence through different mutations [67,72], the emergence of mutationally acquired antibiotic resistance in bacteria [73], such as in the evolution of β-lactamase active sites [74], or the spectrum of mutations causing cancer in humans [75].

## Methods

### Bacteria strains, media, and culture conditions

The *E*. *coli* K12 strain MC4100 [54], which is Cyc^S^ and salicin-negative (Sal^−^) and has an *araD139* (Ara^-^) mutation, was used in this study. We also used a set of 6 *E*. *coli* strains CC101-106 [52]. Each CC strain is Lac^−^ and contains a different point mutation in *lacZ* affecting residue 416 in β-galactosidase. The mutations necessary for Lac^+^ reversion are AT→GC (CC106), GC→AT (CC102), GC→TA (CC104), GC→CG (CC103), AT→TA (CC105), and AT→CG (CC101).

“Unlimited” media used for exponential batch culture was the minimal medium previously described [76], supplemented with 0.2% glucose. In all chemostat cultures, the dilution rate was set at 0.1 h^−1^ [76]. We used the following media for 80 ml chemostat cultures, resulting in 5 different nutritional states. In the glucose-limited (C-limited) medium, the minimal medium was supplemented with 0.02% glucose. For O-limited chemostats, the airflow through the sparger was blocked to create an oxygen-depleted environment and supplemented with 0.08% glucose as described previously [77]. An Fe-depleted environment was obtained by removing sodium citrate and Fe_2_SO_4_ from the medium as described in [76] and supplemented with 0.04% glucose. In addition, contaminating iron was removed from all glassware and other chemostat equipment by soaking for 24 h in 2% hydrochloric acid followed by rinsing with MilliQ water. The P-limited medium contained 0.2% glucose and was as previously described [78]. The N-limited medium was supplemented with 0.2% glucose and contained the same basal medium as the P-limited culture but with (NH_4_)_2_SO_4_ reduced to 0.03 g/l and 1 mM K_2_HPO_4_. The higher glucose addition in some media was to ensure glucose excess in all conditions except under carbon limitation. Anaerobic, phosphate-, nitrogen-, and iron-limiting conditions result in less respiration and more fermentative metabolism, which consumes more glucose, hence the need for more glucose in the media to avoid a double limitation.

### Isolation of mutants on selective media

Acquisition of resistance to cycloserine from cycloserine-sensitive populations of MC4100 (Cyc^S^→Cyc^R^ assay) was used to select mutations in *cycA*. The cycloserine-sensitive *E*. *coli* was grown for 72 h in chemostats at a growth rate (dilution rate) of 0.1 h^−1^ in different nutrient-limited chemostats before plating culture samples on cycloserine plates for detection of Cyc^R^ mutants. The cycloserine plates consisted of 0.2% wt/vol glucose, 40 μM cycloserine (Sigma-Aldrich), and 1.5% wt/vol agar in minimal medium A.

For the Lac^+^ reversion assay, bacteria containing mutant *lacZ* genes were grown in the same growth conditions as used for the detection of Cyc^R^ for 72 h in chemostats. The Lac^+^ reversion assay was performed by mixing 1 ml of concentrated cultures containing 1.3–2.3 x 10^10^ cells with 8 ml of molten (45°C) MMA-top agar (0.7% agar in MMA) in a 50-ml tube. The contents were then overlaid onto 3 separate lactose MMA agar plates, which contain 1.5% wt/vol lactose as a sole carbon source. The total number of Lac^+^ colonies in each sample was counted after 48 h incubation at 37°C.

We used the reversion of the arabinose-negative MC4100 (with a frameshift mutation in *araD*) to arabinose positive (Ara^−^ → Ara^+^) to detect a 1 bp indel in araD by plating cultures on MMA agar plates containing arabinose as the sole carbon source. For the IS transposition mutation rates, we used salicin minimal agar plates; growth on salicin requires a transposition of an IS element as described previously [53]. Arabinose and salicin plates were based on MMA and contained 1% wt/vol arabinose and 1% wt/vol salicin respectively.

### Analysis of mutation rates

We used the Luria-Delbruck fluctuation test [79] for measuring mutation rates in nutrient-unlimited batch cultures but this method cannot be applied to continuous cultures. To determine mutation rates in chemostats, we adopted a method routinely used for continuous cultures [80] as described in detail below.

For the Luria-Delbruck fluctuation tests [79], a single colony of wild-type MC4100 was inoculated in 5 ml MMA medium, supplemented with 0.2% wt/vol glucose and allowed to propagate overnight at 37°C with shaking at 200 rpm. The overnight cultures were diluted in 5 ml fresh MMA medium, supplemented with 0.2% wt/vol glucose and allowed to grow to optical density at 600 nm to 0.6. The exponentially growing cultures were then diluted 10,000-fold into 100 ml in MMA medium supplemented with 0.2% wt/vol glucose and divided equally into 20 separate McCartney bottles, each receiving 5 ml. The freshly diluted cultures were then incubated at 37°C overnight with shaking at 200 rpm. Culture samples were then plated separately on cycloserine, arabinose, and salicin plates. The plates were then incubated for 24 h at 37°C to detect Cyc^R^ mutant colonies and 48 h for detection of Lac^+^, Ara^+^, and Bgl^+^ mutants, respectively. For total colony forming unit counts, aliquots of appropriately diluted cultures were plated on LB-agar plates. The mutation rates were then estimated from the number of resistant colonies per culture and total cell count by using the Ma-Sandri-Sarkar maximum likelihood (MSS-MLL) analysis [79].

For mutation rates in chemostats, a single colony of MC4100 was inoculated in 5 ml of the above 5 nutrient-limited minimal media for 6 h; 1 ml of this starter culture was used as inoculum for chemostats. Culture samples were taken at 2 time points, at time 0 h (i.e., when cultures reached a steady OD_600_ (0.2 to 0.3, equivalent to 1.3–2.3 x 10^10^ bacteria in 80 ml depending on the chemostat composition) and 1 sample at 72 h at the same maintained density. Aliquots were then plated or overlaid separately on the cycloserine, lactose, arabinose, and salicin plates described above as well as for total counts on LB agar. Mutation rates were calculated by using the following equation [80]: *μ* = [1(r_2_ –r_1_)]/[*N*λ(t_2_—t_1_)], where, r_1_ and r_2_ are the number of mutants detected at times t_1_ and t_2_ respectively; *N* is total cell number, which remains constant in a chemostat but was determined in each sample; λ is dilution rate, which was set at 0.1 h^−1^ in these experiments. By adjusting population sizes to account for mutation rate differences, we aimed to obtain a mean of 100 colonies per plate for counting.

### Protein and gene expression analyses

The levels of RpoD, RpoS, DinB, and MutS proteins in chemostats and exponentially growing cultures of *E*. *coli* were determined by using the western blot protocol described previously [50]. The expression level of *mutY* was analysed in the 6 different nutritional conditions as described for the *E*. *coli* MC4100 derivative strain BW3500 containing a *mutY-lacZ* transcriptional fusion [46].

### Determination of the mutational spectrum in *cycA*

The mutation spectrum in *cycA* in Cyc^R^ mutants obtained in the nutritional states was determined by sequencing the entire length of a PCR product of *cycA* in Cyc^R^ mutants. A total of 1,399 Cyc^R^ mutants isolated from 6 replicate cultures from each condition (in total 249 from Fe-limited, 228 from Un-limited, 234 from O-limited, 240 from N-limited, 203 from C-limited, and 245 from P-limited cultures) were randomly chosen for PCR sequencing as described in [30].

The identified mutations were categorized into BPSs, SIs, LIs, and transpositions involving one of several (IS) elements. Rates for each class and each individual type of mutation were estimated from their frequency amongst the sequenced Cyc^R^ mutants and the overall mutation rate for the *cycA* gene based on the fluctuation tests and chemostat methods described above. We excluded only 1 culture from the analysis (Fe-limited) in which a jackpot event resulted in 75% of mutations of the same sequence.

### Analyses of mutant fitness

The fitness of Cyc^R^, Lac^+^, Ara^+^, and Bgl^+^ mutants relative ancestral MC4100 in the same 6 conditions in which they were isolated was analysed as described previously in [40]. Fitness of Cyc^R^, Lac^+^, Ara^+^, and Bgl^+^ in the nutrient-unlimited condition was obtained by comparing the exponential growth rate of mutants with that of wild-type *E*. *coli*.

### Cluster analysis

The tree showing the relationship among mutational spectra from the 6 different environments was based on the PAST software package [44].

### Statistical analysis

The upper and lower limits with 95% confidence interval of mutation rates for Cyc^S^>Cyc^R^, Ara^−^>Ara^+^, and Bgl^-^>Bgl^+^ were determined by the FALCOR web tool [79]. Student *t* test was performed using Microsoft Excel. In all *t* tests, we used the 2-tailed test, assuming 2-sample unequal variance. SDs among replicates are shown in the text and were also calculated using Microsoft Excel. The SD was based on 20 replicate cultures for the Luria-Delbruck experiments and at least 6 replicate chemostats in each environment.

## Supporting information

S1 Table Sequence changes in 6 conditions. (XLSX)

S1 Data Numerical data in figures. (XLSX)

## Abbreviations

bp: base pair
BPS: base-pair substitution
C: carbon
Cyc^S^: cycloserine sensitivity
Cyc^R^: cycloserine resistance
Fe: iron
IS: insertion sequence
LDR1: a deletion of 12 bp at base positions 96–108 of *cycA*
LDR2: a deletion of 18 bp in the 918–948 region of *cycA*
LI: deletion and insertion indels >1bp
LIR1: an insertion of 12 bp at base positions 96–108 of *cycA*
LIR2: an insertion of 18 bp in the 918–948 region of *cycA*
MA: mutation accumulation
MMR: mismatch repair
N: nitrogen
ns: not significant
O: oxygen
other-IS: other insertion sequences
other-LIs: other deletion and insertion indels >1bp
P: phosphorous
ppGpp: guanosine tetraphosphate
SI: single base-pair indels
SIM: stress-induced mutagenesis
Ti/Tv: transition to transversion
Un: nutrient-unlimited
UPGMA: unweighted pair-group method with arithmetic mean
*μ*_BPS_: BPS mutation rate
*μ*_C>A_: C to A mutation rate
*μ*_IS_: IS mutation rate
*μ*_LI_: LI rate
*μ*_SI_: SI mutation rate
*μ*_X_: rates of the various types of mutation
*μ*_TOTAL_: total mutation rate
